# Effect of aging on the female reproductive function

**DOI:** 10.1186/s40834-017-0050-9

**Published:** 2017-10-03

**Authors:** Koumei Shirasuna, Hisataka Iwata

**Affiliations:** Laboratory of Animal Reproduction, Department of Animal Science, Tokyo University of Agriculture, 1737 Funako, Atsugi, Kanagawa 243-0034 Japan

**Keywords:** Senescence, Aging, Corpus Luteum, Oviduct, Uterus, Immune cells

## Abstract

Aging is a complex biological process that involves the accrual of bodily changes over a long life span. In humans, advanced maternal age is associated with infertility and adverse pregnancy complications. Cellular and organic senescence is hypothesized to contribute to the age-related decline in reproductive function. Accumulating evidence suggests that immune cells play pivotal roles in physiological reproductive function and pregnancy. The concept of “inflammaging” has recently emerged- an age-dependent, low-grade, chronic, and systemic inflammatory state induced by the senescence-associated secretory phenotype (SASP), which is produced by the innate immune, parenchymal, and nonparenchymal cells within the organs. In the present review, we discuss how cellular senescence and inflammaging accelerate reproductive failure in women by promoting SASP and immune-senescence during the establishment of pregnancy. In addition, we discuss the role of immune cells and their senescence in reproductive function, particularly in the ovaries (the corpus luteum), oviduct, and uterus.

## Background

Modern social and lifestyle changes have led to an increased number of women who conceive in their late 30s, resulting in an overall higher conception age than before. Women who conceive at an older age have a higher risk of preterm birth and pregnancy-induced hypertension syndrome [1]. It has recently been understood that the quality of fertility (rate of pregnancy) drastically decreases with the qualitative deterioration of oocytes due to aging. In older women, for example, the oocytes may contain abnormal chromosome division, decreased mitochondrial quality, including the accumulation of mutations in the mitochondrial DNA and low ATP production, increased oxidative stress, and decreased antioxidant levels [1–3]. Combined with an increased risk of spontaneous pregnancy loss, such abnormalities may also be associated with a higher chance of early pregnancy loss after in vitro fertilization (IVF). Therefore, aging plays a critical role in fertility. However, does a decline in fertility with aging depend only on the aging of oocytes? It has been reported that despite using high-quality embryos in case of artificial insemination, embryo transfer, and IVF, the pregnancy success rate falls below 50% for cows [4, 5]. Because it is possible that reproductive function and the system (the ovaries, oviduct, and uterus) are affected by maternal aging, it is important to ascertain the reproductive health of a prospective mother in addition to qualitatively assessing the oocytes for ensuring the success of pregnancy.

It is well known that diseases and infections play a more significant role in death than senescence. In addition to causing direct mortality, pathogenic infections indirectly cause death by affecting the immune function and reducing the resistance to an infection with age [6]. In many mammals, including humans, T cell aging initially occurs owing to thymus atrophy caused by aging [7]. Although the number of innate immune system cells such as neutrophils and macrophages does not decrease, over time, the function of such cell (e.g., phagocytosis) decreases with age [8]. Considering the acquired immune system, the peripheral blood leukocyte number decreases with age in cows [9], and their susceptibility infectious diseases such as mastitis and endometritis also increases [10]. Because the immune system plays an essential role in the defense of the body, it is also known to contribute to the regulation of reproductive function and pregnancy. From the perspective of the maternal immune system, a conceptus is a semi-allogeneic tissue that must be rejected; therefore, the evident question is why does that not happen? Research has shown that the immune system and immunological processes are involved in an array of processes beyond fetal immunotolerance, including preparation of the endometrium for implantation of the blastocyst and maintenance of pregnancy and parturition [11]. CD4 + CD25 + Foxp3+ regulatory T cells (Treg cells) play a vital role in regulating immune tolerance at the implantation site to support implantation and successful pregnancy in mice and humans [12, 13]. Disruption of these well-controlled immune functions can lead to infectious diseases, infertility, and numerous pregnancy complications.

Here, we discuss the relationship between reproductive tissue, including the ovaries (the corpus luteum), oviduct, and uterus, and aging, along with immune senescence. For this review, we referred to published data on humans, rodents, and ruminants, especially cows. Research on human reproduction is limited owing to the difficulties involved in investigating biological samples from women. Therefore, it has been proposed that a bovine model may be suitable for studying the reproductive function and age-associated decline in fertility in women because the physiology, including ovarian function, is similar and greater longevity [14–17].

### Aging and luteal function

**Fig. 1:**
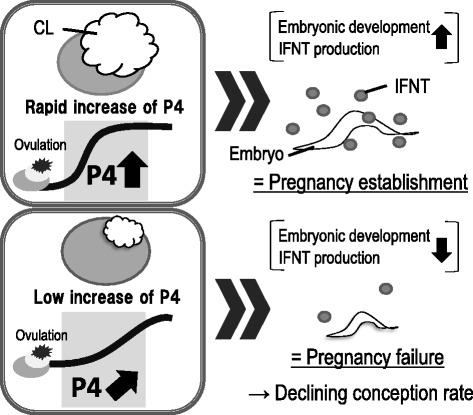
Relationships between increase of P4 levels after ovulation, embryonic development, and IFNT production. There is a close association between poor embryonic development with low IFNT production and the subsequent low P4 levels in cows. Furthermore, P4 supplementation during the early luteal phase results in an improved embryonic development and increased IFNT production. These findings indicate that one of the key hormones in the establishment of pregnancy and the control of embryonic development is the CL secreted P4

The corpus luteum (CL) is a unique, transient endocrine structure with well-coordinated mechanisms by which its development, maintenance, and regression are effectively controlled. The main function of the CL is to produce progesterone (P4), which is necessary for the establishment and maintenance of pregnancy [18]. Rapid development of bovine CL occurs within 8–10 days after ovulation, which is accompanied by active angiogenesis and vascularization of the preovulatory follicle. Increasing P4 concentrations after ovulation enhance conceptus elongation, while lower P4 levels during the early luteal phase delay embryonic development [19]. When pregnancy is established, interferon tau (IFNT), a well-known pregnancy recognition signal for the maintenance of CL in ruminants, is secreted by the embryonic trophoblast cells [20]. In cows, IFNT indirectly maintains the CL by attenuating the luteolytic pulses of uterine prostaglandin F_2α_ beginning on approximately day 16 after conception [21]. In particular, there is a close association between poor embryonic development with low IFNT production and the subsequently low P4 levels in cows (Fig. 1). Furthermore, P4 supplementation during the early luteal phase results in improved embryonic development and increased IFNT production [19]. These findings indicate that one of the key hormones in the establishment of pregnancy and control of embryonic development is P4 secreted from the CL [22]. In addition, Lissauer et al. showed that P4 regulates and induces the reduction in the pro-inflammatory cytokines (IFNG and tumor necrosis factor-α) and anti-inflammatory cytokine (IL-10 and IL-5) as well as the increase in IL-4 production on T cells. They suggested the importance of P4 to induce fetal tolerance within the uterus [23].

Malhi et al. [15] analyzed the age-related changes in follicular, luteal, and endocrine function in cows approaching reproductive senescence in order to validate the existing bovine model studying the reproductive events in women approaching menopause. They found that the luteal P4 concentrations and CL diameter were lower in older cows (13–14 years old) than in the younger cows (1–4 years old) [15]. Short telomeres and low telomerase activity are observed in granulosa cells (which differentiate into luteal cells after ovulation) in women (aged over than 37 years old) [24]. These data suggest that both functional and structural degradation of the luteal capabilities occurs owing to aging. Moreover, Erickson et al. [25] reported that the incidence of lutein cyst appears to increase with age in cows, and accounts for more than 50% in non-pregnant older cows. However, Hasler et al. [26] have reported that aging does not affect pregnancy rate with respect to embryonic transfer in cows. Although proper functioning of the CL is obviously essential for the establishment of pregnancy, few studies have examined the effect of aging specifically on the luteal function and subsequent pregnancy establishment, which is why further studies are needed to clarify these points.

### Aging and oviduct function

The oviduct is the essential site for the transport of gametes, and is the platform for fertilization and regulation of early embryonic development, thereby determining the success of pregnancy [27]. The transport of gametes and embryo along the oviduct is regulated by active motion via the contraction and relaxation generated by the ciliary beating of the oviduct epithelial cells (OECs) [28]. OECs also secrete various types of cytokines and growth factors that regulate embryonic transportation and development [27]. The oviduct is also crucial for creating a suitable microenvironment for fertilization and early embryogenesis; therefore, unregulated or pathological conditions in the oviduct may directly lead to infertility.

Limited information is available regarding the age-dependent changes influencing the functioning of the oviduct. Yan et al. [29] have reported on the morphological and functional differences in the gap junctions of oviduct epithelia in young and adult hamsters. We have previously shown that the age-dependent changes in bovine OECs are mediated in part by pro-inflammatory signaling and extracellular matrix (ECM)-related components [30]. In particular, several proinflammatory cytokines, including interleukin (IL)-1β, IL-1α, IL-17C, IL-8, S100A8, S100A9, and tumor necrosis factor-α, were more active in aged OECs than those in young OECs. Among these, IL-1β is involved in pregnancy complications, such as infertility, early pregnancy loss, and preeclampsia [31–33]. In addition to the age-dependent increase in IL-1β, IL-1β treatment increases in the levels of senescence-associated β-galactosidase activity and p21 protein expression, which are widely known as markers of senescence, thus indicating that IL-1β induces cellular senescence [34]. These findings suggest that aged bovine OECs secrete IL-1β, which further stimulates oviduct cell senescence and inflammatory responses in an age-dependent manner.

In contrast to the upregulated proinflammatory cytokines and their genes, several of the major genes in aged OECs that are associated with ECM-related factors such as collagens, decorin, periostin, biglycan, and lumican are downregulated [30]. It is known that ECM components decline with age, which results in decreased elasticity and increased stiffness of the epithelial tissues [35–37]. Furthermore, aged bovine OECs express higher levels of F-actin cytoskeleton, and their functional capabilities, including villous movement and cell proliferation, are lower than those of the young OECs, which play a role in the age-dependent dysfunction in actin polymerization and depolymerization in OECs [30]. Therefore, the downregulation of ECM-related factors may be responsible for the development of age-dependent impairment of the functions of the bovine oviduct.

### Aging and uterine function

The uterus is the essential organ for pregnancy that provides the architecture for embryonic development, elongation, implantation, and placentation, in addition to fetal development. In contrast to the ample evidence of the negative effect of aging on oocytes, aging’s impact on uterine function is less clear. It has been reported that the age of female recipients does not influence implantation rates with respect to embryonic transfer, indicating that the uterus is able to support pregnancy even at older ages [38]. On the other hand. Concerning mares, old age (over 15 years) is associated with increased accumulation of inflammatory cells within the endometrium, reduced pregnancy rate, and increased chance of pregnancy loss when compared with younger mares (5–7 years old) [39].

The uterus does show senescence considering that the uterine tissue derived from older women (over 45 years old) shows increased cellular senescence (existence of senescence-associated β-galactosidase positive cells) than that belonging to younger women (less than 45 years old) [40]. Simmen et al. [41] reported that aging of the uteri leads to the downregulation of several genes associated with cell proliferation in mice, indicating the presence of senescent cells with impaired proliferation owing to uterine aging. Uterine dysfunction, senescence, and contractile activity in the myometrium of younger rats are significantly higher than that of older rats [42]. Moreover, immune- and inflammatory response-related pathways are upregulated in relation to the age of the rat uterine horns, as seen by microarray analysis [42]. In addition, we recently observed that inflammation related- and interferon-signaling pathways were more active in older bovine endometrial cells than those in younger cells [43]. We, therefore, suggested that DNA damage is accumulated and cell proliferation ceases as the uterus ages. We also hypothesized that age-dependent changes in the bovine endometrial cells are partly mediated by pro-inflammatory signaling, interferon signaling, and cell cycle- related components [43].

Women of older age are more likely to experience pregnancy complications and diseases, such as miscarriage, low birthweight, preterm or postterm delivery, and caesarean delivery. Cano et al. [38] reported that the implantation rates were similar for recipient women younger and older than 40 years with respect to IVF-ET, but higher rates of pregnancy loss were observed for the older women; the transfer of embryos was found to be similar in number and quality. As this mechanism, they found the secretion of estradiol and P4 by the placenta started earlier in pregnancies included in the group <40 years that in women >40 years [38]. This retardation of steroid synthesis in the older women was associated with increase in pregnancy loss [38]. These findings suggest that uterine aging may be a factor influencing the poor reproductive performance of older women. Similarly, Pellicer et al. [44]. reported that a significant increase in estradiol concentrations (which probably represents the luteo-placental shift) was detected 2 weeks earlier in younger than in older patients, suggesting a defective vasculature of the uterus that could ultimately be the cause of an increased abortion rate in patients aged >40 years. They concluded that senescence affects both the ovary and the uterus [44].

In cows, the frequency and intensity of adenomyosis, which is closely related to endometriosis, increases with age and potentially interferes with the reproductive processes [45]. Hirota et al. [46] have shown that the uterine-specific p53 deficiency confers uterine senescence, resulting in the promotion of preterm birth in mice. A treatment with a combination of rapamycin (an mTORC1 inhibitor) and P4 counters the spontaneous preterm birth due to premature decidual senescence in the mice with the uterine-specific p53 deficiency. These studies collectively suggest that uterine capability necessary to establish and maintain a pregnancy may change in relation to aging and senescence, and excessive senescence in the uterus may directly lead to infertility and reproductive complications.

### Importance of immune cells in pregnancy

In general, the immune system is categorized as innate and acquired immune system. The representative cells of innate immune cells are neutrophils and macrophages. When bacteria invade or tissues are damaged, innate immune cells rapidly accumulate at the affected site and initiate an inflammatory response. Lymphocytes such as T cells and B cells belong to the acquired immune system, which plays a role in eliminating pathogens by creating an immunological memory of the infection sources. How does the immune system influence the establishment of pregnancy?

Pregnancy is initiated by the fusion of the sperm with the egg and is established when the developed embryo is implanted within the mother’s uterus. Although the fertilized egg comprises genetic material from both the parents, the mother’s immune system must accept it despite it being semi-allograft or semi-foreign in nature. This phenomenon is in conflict with the mother’s immune function of eliminating foreign material from the body. Therefore, it is thought that a special “immune tolerance for pregnancy works in response to pregnancy”.

**Fig. 2:**
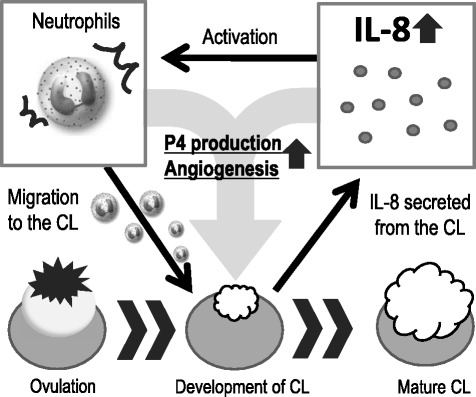
Possible model of inflammatory-like luteal development. The mechanism of luteal development is considered as an inflammation-like response involving many types of immune cells such as neutrophils and macrophage. They participate in the luteal development by increasing angiogenesis and P4 production

After ovulation, numerous leukocytes are translocated to the luteinizing theca area of the developing CL [47]. The series of events from ovulation to luteal development involves bleeding, immune cell infiltration, tissue remodeling and angiogenesis, which implies that the development of the CL following ovulation is a physiological injury involving an inflammatory response. Indeed, a considerable number of immune cells (neutrophils and macrophages) and high levels of IL-8 (a chemoattractant) are seen during the early luteal phase in the CL of cows [48–50] (Fig. 2). In an in vitro study, co-culture of luteal or granulosa cells with neutrophils or macrophages, increased P4 production [51]. The early CL induces neutrophil migration in vitro by secreting IL-8, and neutrophils and IL-8 induce angiogenesis in vivo [52, 53] and in vitro (by the formation of capillary-like structures) [48, 54]. On the other hand, Turner et al. [55] have reported on the importance of macrophages in maintaining vascular integrity in the CL. Progressive macrophage elimination is associated with ovarian hemorrhage and reduced P4 levels, which negatively affect the luteal tissue owing to significant endothelial cell depletion and increased erythrocytes, thereby resulting in infertility [55]. Thus, immune cells such as neutrophils and macrophages have key roles in the development of the CL and ensuring pregnancy success.

Various immune responses occur when an embryo is implanted within the uterus. Immune cells such as macrophages and uterine NK cells accumulate in the uterus and promote embryonic growth and implantation [56]. The immune cells considered the most important for the immune tolerance of pregnancy are the Treg cells [57, 58]. Treg cell numbers increase in the peripheral blood, spleen, and lymph nodes of pregnant humans and mice. The Treg cells also continuously regulate production of anti-inflammatory cytokine IL-10. Depletion of Treg cells during early pregnancy leads to implantation failure in mice, whereas supplementation with Treg cells reduces the incidence of miscarriage in mice [13]. The population of Treg cells is low in women with preeclampsia and recurrent pregnancy loss, which results in an induction of inflammatory responses associated with the occurrence of such conditions [59]. In cows, γδT cells may have an inhibitory function and may be involved in immune tolerance and pathogenesis with respect to pregnancy [60]. Although further research is underway, it is clear that the immune system plays an important role in implantation and pregnancy.

### Possible role of immune-senescence in pregnancy

In humans, the susceptibility to various diseases such as metabolic syndrome (hypertension and arteriosclerosis), cancer, and infection increases with age. Because these diseases are commonly found among many elderly people, they can be regarded as a part of the aging phenotype. It is known that the immune cells play a critical role in the regulation of pregnancy as well as in the onset of these diseases. Therefore, it is important to understand the phenomenon of immune-senescence associated with aging in order to achieve improved pregnancy rates and prognoses.

T cells are the most affected by aging in humans and cows. The T cells are derived from the differentiation and maturation of progenitor cells in the bone marrow in the thymus, which sharply regresses with age [7]. That is, it is difficult to produce T cells specific to antigens such as new types of viruses and pathogenic bacteria as a person ages [61]. As discussed earlier, Treg cells are also important with respect to pregnancy, but even their function is affected by aging. In particular, it is thought that inducible Treg cells produced in the thymus decrease with age and thereby weaken the regulation of cellular functions of the inflammatory system and cytokine production [61]. Given that such age-related changes also affect pregnancy, the capability of immune tolerance to pregnancy and acceptance of embryos is reduced and excessive inflammation is induced, leading to poor implantation and pregnancy failure. On the other hand, reduced number of T cells accompanying aging is not noticeable for the cell types of the innate immune system (neutrophils and macrophages), but it is known that their function (phagocytosis) is affected by aging.

Recently, it has been widely recognized that physiological or pathophysiological aging can be driven by proinflammatory cytokines such as IL-1β, IL-6, IL-8, and tumor necrosis factor-α, leading to the development of senescence-associated secretory phenotype (SASP), which is produced by the innate immune, parenchymal, and nonparenchymal cells [61–64]. Thus, the concept of “inflammaging” has emerged, which is characterized by an age-dependent, low-grade, chronic, and systemic inflammatory state [64]. Indeed, the plasma concentrations of various SASP cytokines show age-dependent increases in healthy humans [65] and diseases, including lifestyle diseases and rheumatoid arthritis, are relevant to the concept of inflammaging. Therefore, inflammaging in immune cells and reproduction-related cells may have a potentially detrimental impact on pregnancy due to the dysfunction of immune tolerance. In the future, it is necessary to clarify the mechanisms of senescence and inflammatory changes in reproductive and immune cells that are important for the establishment of pregnancy.

## Conclusion

Aging is the result of complex interactions involving biological, physical, and biochemical processes that cause dysfunctions in cells and organs. Owing to the biologically unavoidable fate of aging, the functions of cells of the ovarian, oviduct, uterine and immune systems are affected. Cellular senescence may contribute to the progressive decline in female reproductive capability by decreasing the quality of oocytes, dysregulating reproductive tissues such as the CL, oviduct, and uterus, and increasing complications during pregnancy. It will be helpful to investigate further the impact of senescent cells and inflammaging on reproduction, and it may be possible to achieve efficient regulation of pregnancy and in order to overcome the decline in fertility associated with aging.
